# The role of immunoglobulin in cerebrospinal fluid on the differential diagnosis of autoimmune encephalitis and viral encephalitis in children

**DOI:** 10.1186/s12887-024-04824-w

**Published:** 2024-06-08

**Authors:** Xiaolu Hu, Shen Cheng

**Affiliations:** 1 Clinical Laboratory, Jiangxi Province Children’s Hospital, Nanchang, Jiangxi 330000 China; 2 Pediatrics, The First Affiliated Hospital of Zhejiang Chinese Medical University (Zhejiang Provincial Hospital of Traditional Chinese Medicine), Hangzhou, 310000 China

**Keywords:** Cerebrospinal fluid, Immunoglobulin, Autoimmune encephalitis in children

## Abstract

**Background:**

A case-control study was conducted to analyze the role of cerebrospinal fluid immunoglobulin in the differential diagnosis of autoimmune encephalitis and viral encephalitis in children.

**Methods:**

One hundred and twenty patients with autoimmune encephalitis (AE) treated in our hospital from February 2021 to February 2022 were included as the observation group (AE group). 100 patients with viral encephalitis (VE group) were selected as the control group. The clinical data of all patients were collected and analyzed retrospectively. Immunoglobulin G (IgG) and immunoglobulin A (IgA)in cerebrospinal fluid of the two patients were measured by immune turbidimetry. Immunoglobulin M (IgM), and the diagnostic value of immunoglobulin in cerebrospinal fluid (CSF) in patients with AE was analyzed by receiver working curve (ROC).

**Results:**

The level of IgG in the cerebrospinal fluid of the AE group was higher than that of the VE group, and the level of IgM was lower than that of the VE group, and the difference was statistically significant (*P* < 0.05). There was no significant difference in IgA levels between the two groups (*P* > 0.05). In terms of Magnetic Resonance (MR) features, the paraventricular, hippocampal, occipital and parietal lobes were more involved in AE patients, frontal and temporal lobes were more involved in VE patients, and paraventricular and occipital lobes were involved in MS. The proportion of bilateral extensive lesions in both groups was significantly higher than 50%. The proportions of patients in the AE group involving the lateral ventricle, insula, and parietal lobes were significantly higher than those in the VE group, and the proportions involving the basal ganglia, temporal lobes, and frontal lobes were significantly lower than those in the VE group, and the differences were statistically significant (All *P* < 0.05). The Area Under Curve (AUC) of IgG, IgA and IgM alone in the diagnosis of AE were 0.795(0.587–0.762), 0.602(0.502–0.631) and 0.627(0.534–0.708), respectively with the sensitivity values of 81.24% and 65.608, respectively and the specificity values of 65.08%, 57.54% and 75.01% respectively. The AUC of IgA + IgM in the diagnosis of AE was 0.733(0.617–0.849), and the sensitivity and specificity are 62.58% and 75.07% respectively. The AUC of IgA + IgG in the diagnosis of AE was 0.823(0.730–0.917), and the sensitivity and specificity were 81.24% and 67.54% respectively. The AUC of IgG + IgM in the diagnosis of AE was 0.886(0.814 ~ 0.958), and the sensitivity and specificity were 84.48% and 77.59% respectively. The AUC of IgA + IgM + IgG in the diagnosis of AE was 0.924 (0.868–0.981) with the sensitivity of 93.82%, and the specificity of 77.56%.

**Conclusion:**

The level of immunoglobulin in cerebrospinal fluid can be used as an effective reference index for the diagnosis of AE. The combined detection of IgA, IgM and IgG can improve the accuracy, sensitivity and specificity of AE.

## Background

Encephalitis is the most common central nervous system disease in childhood. It is an inflammation of brain parenchyma caused by different pathogens, which can lead to different degrees of neurological dysfunction [1]. The main clinical manifestations of the patients are fever, convulsion, change of consciousness, focal nerve injury and so on, accompanied by abnormal cerebrospinal fluid and changes of Magnetic Resonance Imaging (MRI) signal. At present, the most common encephalitis in children is viral encephalitis (VE) and autoimmune encephalitis (AE). Previous studies have suggested that viral infection is the main factor causing encephalitis, accounting for about 20% to 50% of all encephalitis with a clear etiology [2, 3]. However, since the first case of anti-NMDA receptor encephalitis was reported in 2007, with the innovation of antibody detection methods, the incidence of AE has increased year by year. In recent years, autoimmune factors have surpassed viruses as the main cause of encephalitis [4, 5].

Immunotherapy is currently the main method for the treatment of AE, but the optimal treatment plan still needs clinical practice [6, 7], delaying the initial immunotherapy will lead to more severe cognitive impairment and increase the risk of poor disease prognosis [8]. Due to the low sensitivity of antibody detection and the lack of specific biomarkers in the early stages of the disease, a large number of AEs cannot be diagnosed definitively. In addition, the high degree of similarity in the clinical presentation of neurological diseases and the lack of expressiveness in children add to the difficulty of early diagnosis and treatment, which also contribute to the poor prognosis of children with encephalitis. Furthermore, even with the application of various tests, the cause of encephalitis remains unknown in 37%-62% of patients, and the probability of mortality and serious sequelae is greatly increased in this group of patients with unknown causes [9, 10]. In addition, the early clinical presentation of autoimmune encephalitis and infectious encephalitis, particularly viral encephalitis, is very similar. More specific clinical indicators are therefore needed to identify viral encephalitis and autoimmune encephalitis in order to improve prognosis and reduce mortality [5, 11, 12].

Peripheral blood immunoglobulin can diffuse into the central nervous system (CNS) through the blood-brain barrier (BBB), achieving relative balance, and maintaining a certain homeostasis [13, 14]. However, due to the presence of BBB, the filtration efficiency of antibodies from serum is very low with the ratio of antibodies in cerebrospinal fluid to serum of about 200-1-500 [15, 16]. However, there are three main reasons for the increase of immunoglobulin in CNS during inflammation. Firstly, the increase of immunoglobulin in peripheral blood during inflammation, which will correspondingly increase the number of CNS diffused into CNS [17]. Then, when inflammation involves CNS, the integrity of BBB is impaired and the entry of peripheral immunoglobulin into CNS is increased. Additionally, peripheral antibody-secreting cells (ASC) can activate and migrate into CNS, or ASC from the local ectopic lymphoid follicle-like structure in CNS synthesizes immunoglobulin, called intrathecal synthesis. Immunoglobulin in cerebrospinal fluid (CSF) is an important index to detect intracranial humoral immunity, which will play an important role in pathophysiology, clinical manifestation and prognosis of many nervous system diseases. Immunoglobulin M (IgM), immunoglobulin G (IgG) and immunoglobulin A (IgA) are commonly used immune indexes in CSF. It has been reported that IgG dominates intracranial humoral immunity in multiple sclerosis (MS) and chronic encephalitis of unknown etiology, and IgM synthesis dominates in tick-borne meningeal polyneuritis Bannwarth [18]. However, most other inflammatory diseases, such as neurosyphilis and herpesvirus encephalitis, which often accompanied by the synthesis of IgA and IgM [19, 20]. These clinical findings suggest that cerebrospinal fluid immunoglobulins (Ig) may be involved in immune responses related to infection and self-antigens. Therefore, the detection of IgM, IgG and IgA in cerebrospinal fluid can play an important role in the diagnosis of central nervous system diseases. However, there are few reports about the role of cerebrospinal fluid immunoglobulin in the diagnosis of autoimmune encephalitis in children, and its specific diagnostic value needs to be further explored. Under this background, it is very necessary to carry out such research. In this paper, 120 patients with AE and 100 patients with VE diagnosed in our hospital from February 2020 to February 2022 were collected. And the levels of immunoglobulin in cerebrospinal fluid of the two groups were compared to explore the diagnostic value of immunoglobulin in cerebrospinal fluid in children with autoimmune encephalitis, so as to provide new ideas for clinical treatment of children with AE.

## Materials and methods

**Fig. 1:**
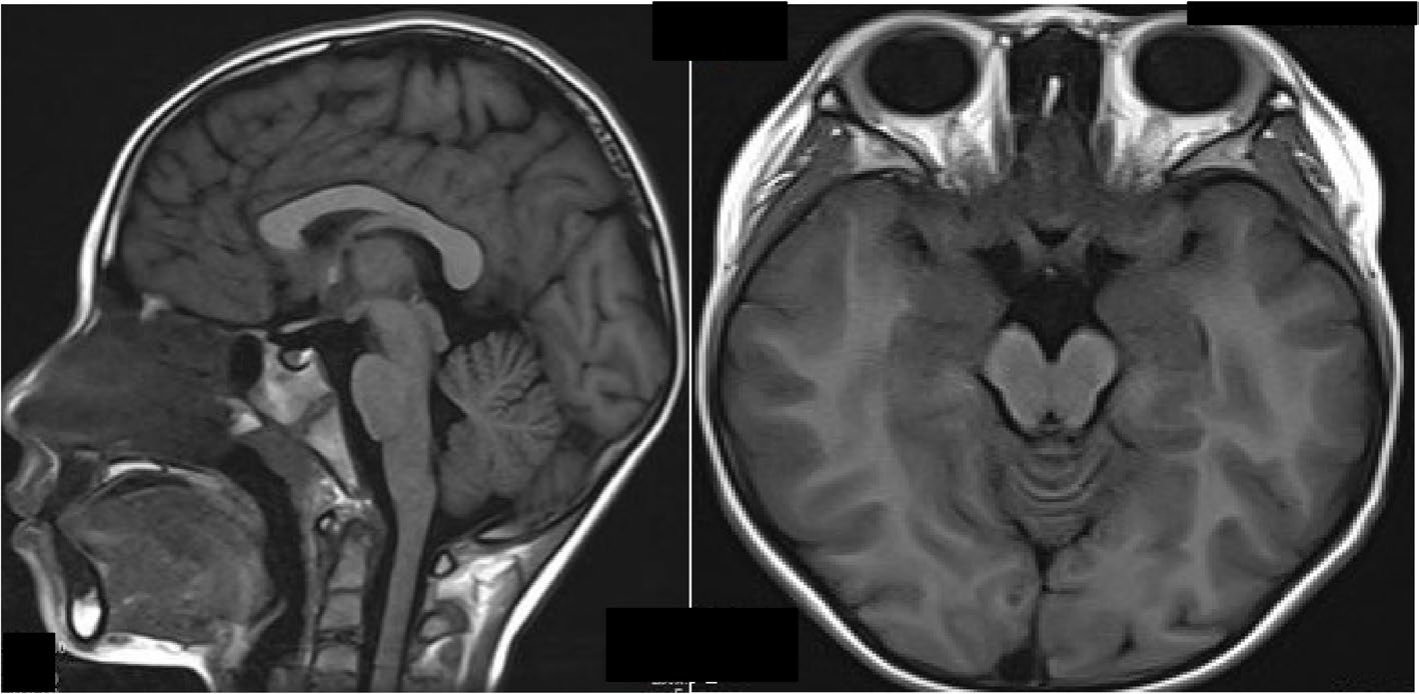
MRI scans of the patients as obtained from the hospital

One hundred and twenty patients with AE treated in our hospital from February 2021 to February 2022 were included as the observation group (AE group). Another 100 patients with viral encephalitis (VE group) were selected as the control group. The clinical data of all patients were collected including MRI scans (Fig. 1) and its results and analyzed retrospectively. The general data of the two groups are shown in Table 1.

**Table 1:** The general data between AE group and VE group

Variable	AE Group(*n* = 120)	VE Group (*n* = 100)	t/χ^2^	*P*
Age (years)	5.27 ± 1.41	5.41 ± 1.37	0.743	>0.05
body mass index (BMI)(kg/m^2^)	23.10 ± 2.54	23.06 ± 2.49	0.117	>0.05
Gender			0.030	>0.05
Male	65 (54.17)	53 (53.00)		
Female	55 (45.83)	47 (47.00)		
Course of disease (days)	12.04 ± 3.87	11.87 ± 4.41	0.304	>0.05
Complicated with allergic diseases (case)	9 (7.50)	6 (6.00)	0.193	>0.05
mRs Score (points)	4.52 ± 1.03	4.37 ± 1.22	0.874	>0.05
Clinical symptoms				
Mental behavior abnormality			0.036	>0.05
Yes	85 (70.83)	72 (72.00)		
None	35 (29.17)	28 (28.00)		
Memory loss			0.070	>0.05
Yes	39 (32.50)	44 (44.00)		
None	81 (67.50)	56 (56.00)		
Language barrier			0.433	>0.05
Yes	21 (17.50)	21 (21.00)		
None	99 (82.50)	79 (79.00)		
Motor disorder			1.782	>0.05
Yes	17 (14.17)	21 (21.00)		
None	103 (85.83)	79 (79.00)		
Sleep disorder			1.248	>0.05
Yes	95 (7917)	85 (85.00)		
None	25 (20.83)	15 (15.00)		
Degree of education			0.015	>0.05
Preschool children	67 (55.83)	55 (55.00)		
Primary school	53 (44.17)	45 (45.00)		
Cerebrospinal fluid immunoglobulin				
IgG(g/L)	35.73 ± 2.21	18.26 ± 0.41	77.912	<0.05
IgA(g/L)	4.55 ± 1.72	4.53 ± 1.41	0.093	>0.05
IgM(g/L)	2.33 ± 0.48	3.25 ± 0.37	15.673	<0.05

Inclusion criteria: (1) all patients in the observation group were clearly diagnosed as autoimmune encephalitis, with reference to the “Chinese expert consensus on the diagnosis and treatment of autoimmune Encephalitis” [21]; the control group was clearly diagnosed as viral meningitis. The diagnostic criteria referred to “Zhufutang practical Pediatrics” (8th edition); (2) patients had clinical manifestations such as seizures or mental disorders; (3) the number of white blood cells in cerebrospinal fluid was higher than normal or high amplitude slow wave could be seen in EEG; (4) brain parenchyma infiltration could be seen by head magnetic resonance imaging; (5) the informed consent form of the test was signed by the patient.

Exclusion criteria: (1) patients with hematological diseases; (2) patients with acute and chronic infection; (3) patients with malignant tumor; (4) patients with other autoimmune diseases; (5) patients with mental diseases; (6) patients with incomplete case data; (7) encephalitis caused by other factors; (8) those who had participated in similar research programs.

### Methods

CSF was collected by lumbar puncture in all patients. IgG, IgA and IgM in CSF were determined by immune scattering turbidimetry. The test was performed strictly according to the instructions. The detection instrument was BN-II Special protein Analyzer (SIEMENS Company).

### Observation index

The levels of IgG, IgA and IgM in cerebrospinal fluid were compared between the two groups. CSF was collected by lumbar puncture and pressure measurements were done after the puncture. CSF was then placed in a sterile test tube and can precipitate by forming insoluble protein salts, resulting in a white cloudy or precipitated. Normal values for CSF albumin were 0.15 to 0.45 g/L.

IgA, IgM, IgG combined diagnosis (three indicators combined diagnosis or two indicators combined diagnosis) AE positive standard was that all indicators were higher than normal values. If one or all of the diagnostic indicators were normal, it was negative. Reference value of laboratory immunoglobulin index in our hospital: 0 to 1 year old: IgA is 0.05 to 0.41 g/L, IgG is 3.2 to 7.2 g/L, and IgM is 0.23 to 0.91 g/L; over 1 year old: IgA is 2 ~ 2.7 g/L, IgG was 11.52 ~ 14.22 g/L, IgM was 0.84 ~ 1.32 g/L.

### Statistical analysis

SPSS22.0 statistical software was used to analyze the test data. The measurement data with normal distribution and uniform variance were expressed by mean ± standard deviation (x ± s), independent sample t-test was used for comparison between groups, and counting data were expressed as cases and percentage, and χ 2 test was used. The receiver operating characteristic curve (ROC) was used to analyze the diagnostic value of immunoglobulin level in cerebrospinal fluid for AE, and the difference was statistically significant (*P* < 0.05).

## Results

### Comparison of general data between AE group and VE group

The general data of patients in the AE group and the VE group, such as age, body mass index, gender, disease course, allergic disease, mRs score, mental and behavioral abnormalities, memory loss, movement disorder, language disorder, sleep disorder and cerebrospinal fluid IgA level were compared. There was no statistical difference in the data (*P* > 0.05). The level of IgG in cerebrospinal fluid of AE group was higher than that of VE group, and the level of IgM was lower than that of VE group (*P* < 0.05). All the results are shown in Table 1.

### The frequency of MR imaging lesions between AE group and VE group

In terms of Magnetic Resonance (MR) features, the paraventricular, hippocampal, occipital and parietal lobes were more involved in AE patients. The frontal and temporal lobes were more involved in VE patients, and paraventricular and occipital lobes were involved in MS. The proportion of bilateral extensive lesions in both groups was significantly higher than 50%. The proportions of patients in the AE group involving the lateral ventricle, insula, and parietal lobes were significantly higher than those in the VE group, and the proportions involving the basal ganglia, temporal lobes, and frontal lobes were significantly lower than those in the VE group, and the differences were statistically significant (All *P* < 0.05). The results are shown in Table 2.

**Table 2:** The frequency of MR imaging lesions in the AE group and the VE group(n/%)

MR		AE Group (*n* = 120)	VE Group (*n* = 100)	χ2	*P*
Spinal cord		0 (0.00)	0 (0.00)	-	-
Paraventricular to lateral ventricle		60 (50.00)	3 (3.00)	58.960	<0.05
Cerebellum		8 (6.67)	6 (6.00)	0.041	>0.05
Insular leaf		53 (44.17)	3 (3.00)	48.715	<0.05
Basal ganglia		15 (12.50)	25 (25.00)	5729	<0.05
Hippocampus		60 (50.00)	38 (38.00)	3.180	>0.05
Occipital lobe		75 (62.50)	53 (53.00)	2.023	>0.05
Parietal lobe		83 (69.17)	19 (19.00)	55.202	<0.05
Temporal lobe		15 (12.50)	56 (56.00)	47.221	<0.05
Frontal lobe		23 (19.17)	69 (69.00)	55.673	<0.05
Bilateral extensive lesions		83 (69.17)	75 (75.00)	0.917	>0.05
Unilateral		8 (6.67)	13 (13.00)	2.534	>0.05

### Diagnostic value of Cerebrospinal fluid Immunoglobulin in AE

**Fig. 2:**
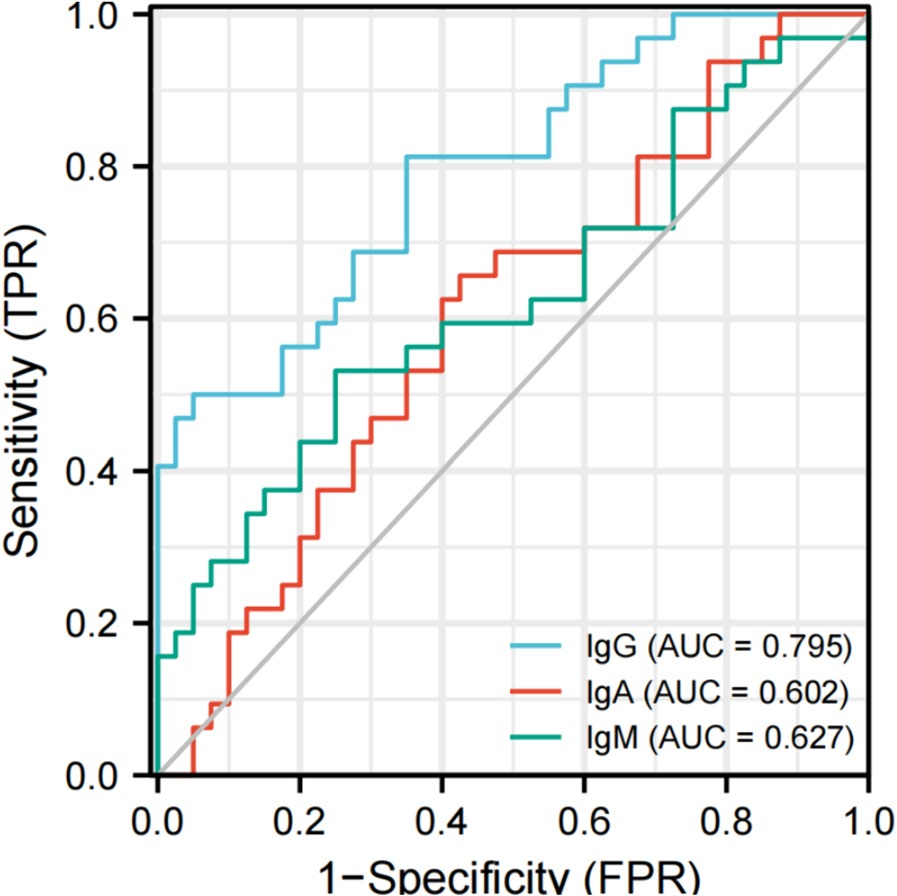
ROC curve of AE diagnosed by IgA, IgM and IgG alone

**Fig. 3:**
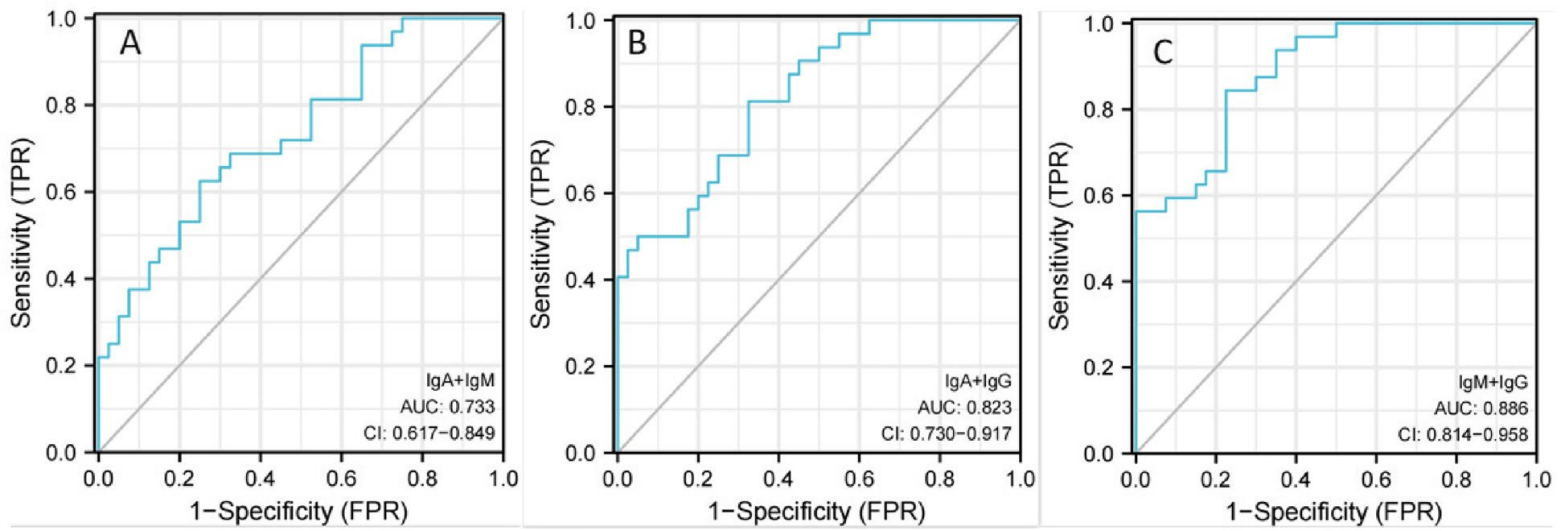
ROC curve of AE diagnosed by IgA, IgM and IgG respectively. Note：A：IgA+ IgM；B：IgA+ IgG；C：IgM+ IgG

**Fig. 4:**
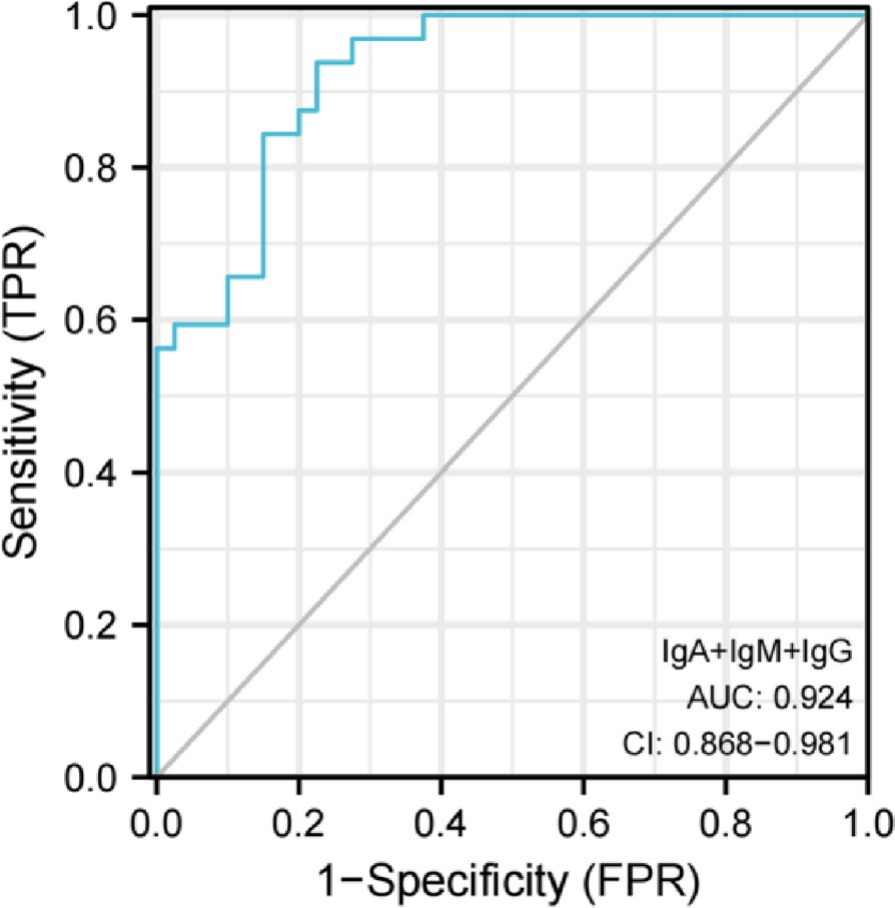
ROC curve of combined diagnosis of AE with IgA, IgM and IgG

The area under the curve (AUC) of IgG, IgA and IgM for the diagnosis of AE were 0.795 (0.587–0.762), 0.602 (0.502–0.631) and 0.627 (0.534–0.708), respectively, with the sensitivity of 81.24%, 65.63% and 53.15%, respectively; and the specificity was 65.08%, 57.54% and 75.01% respectively. The AUC of IgA + IgM in the diagnosis of AE was 0.733(0.617–0.849), and the sensitivity and specificity are 62.58% and 75.07% respectively. The AUC of IgA + IgG in the diagnosis of AE was 0.823(0.730–0.917), and the sensitivity and specificity were 81.24% and 67.54% respectively. The AUC of IgG + IgM in the diagnosis of AE was 0.886(0.814–0.958), and the sensitivity and specificity were 84.48% and 77.59% respectively. The AUC of IgA + IgM + IgG in the diagnosis of AE was 0.924(0.868–0.981), the sensitivity was 93.82%, and the specificity was 77.56%. All the results are shown in Figs. 2, 3 and 4; Table 3.

**Table 3:** Diagnostic efficacy of three indicators of IgA, IgM and IgG for AE alone or in combination

Variable	AUC	sensitivity(%)	Specificity degree(%)	Yoden index	95%CI
IgG	0.795	81.24	65.08	0.462	0.587–0.762
IgA	0.602	65.63	57.54	0.231	0.502–0.631
IgM	0.627	53.15	75.01	0.281	0.534–0.708
IgA + IgM	0.733	62.58	75.07	0.375	0.617–0.849
IgA + IgG	0.823	81.24	67.54	0.488	0.730–0.917
IgG + IgM	0.886	84.48	77.59	0.619	0.814–0.958
IgA + IgM + IgG	0.924	93.82	77.56	0.712	0.868–0.981

## Discussion

AE is a large class of acute or subacute autoimmune diseases caused by immune disorders, with seizures, cognitive impairment, abnormal mental behavior and abnormal limb movement as the main clinical manifestations [22–25]. The research on this disease could be traced back to 1968, when the concept of “marginal encephalitis” was put forward by London doctor Corsellis [25]. He found pathological changes involving the limbic lobe and clinical manifestations such as memory impairment and mental retardation in many cancer patients. In 2007, Dalmau and other female teratoma patients with mental symptoms and cognitive impairment found antibodies against N-methyl-D-aspartate receptor (NMDAR) on the surface of hippocampal neurons, and put forward the concept of “anti-NMDAR encephalitis” [26]. With the development of antibody detection technology, the spectrum of AE antibodies continues to expand, and the etiology and mechanism are gradually revealed. It is found that not all AE are accompanied by tumors, and the lesions are not limited to the limbic system.

Autoimmune encephalitis antibodies can be divided into [27–30], including 1) antibodies against synaptic receptors, such as NMDAR, D2R, AMPAR, GABAAR, GABABR;2) antibodies against ion channels and cell surface proteins, such as LGI1, Caspr2, DPPX, MOG, AQP4;3) antibodies against intracellular antigens, such as Hu, GAD, Ma2. The common non-specific manifestations of AE are mental and behavioral abnormalities, seizures, memory and cognitive disorders, motor disorders, autonomic nervous dysfunction and disturbance of consciousness. Different antibodies often correspond to different clinical phenotypes. For example, NMDAR and D2R are more common in children and youth, while Hu and Ma2 are more common in adults and are more closely related to tumors. In the first symptoms, children often take epilepsy as the first manifestation. Insomnia, seizures, involuntary movements or behavioral changes are more common than adults. Teenagers and adults are more likely to have mental symptoms, including restlessness, hallucinations, delusions and catatonia. Paraneoplastic syndrome is less common in children.

At present, immunotherapy is the main treatment for AE, which is generally divided into first-line immunotherapy, second-line immunotherapy and long-term immuno-maintenance therapy [31]. Timely immunotherapy is associated with a good prognosis. Clinical observation showed that there were bipolar differences in the prognosis of the patients. The mild patients could basically recover to the pre-disease level after the completion of immunotherapy, while the severely ill patients may need to be admitted to the intensive care unit and given mechanical ventilation. After active immunotherapy and long-term rehabilitation training, it is still possible to leave neurological dysfunction or even life-threatening. Some patients may need immunosuppressive therapy for weeks or months as a result of recurrence, and the duration of hospitalization will be greatly extended. With the continuous development and improvement of immunology, many immune antibodies have been successfully found and widely used in clinic, and the etiology of some patients has been identified.

Immune globulin is a key component of the body’s immune system. Previous studies have found that a variety of immune indicators are abnormal in patients with autoimmune encephalitis [32]. In normal CSF, the highest immunoglobulin content is IgG, followed by IgA, and the least is IgM. Immune cells in the CSF can produce IgG, which forms the main antibody to the immunoglobulins in the CSF. As IgG has the smallest molecular weight, it readily crosses the blood-brain barrier; IgM is mainly produced by large monocytes in the spleen and has a relatively large molecular weight, making it difficult to cross the blood-brain barrier. Although IgA has a large molecular weight, which is difficult to penetrate the blood-brain barrier. Immune cells in CSF can produce a certain amount of IgA, therefore, the content of IgA in CSF is more than IgM. When the body is in a normal state, IgG, IgA, and IgM in CSF are easily affected by serum levels. Fan Xiaoying reported for the first time that the levels of IgA, IgM, IgG and other immunoglobulins in the peripheral blood of patients with autoimmune encephalitis were higher than those of the normal control group [33]. Subsequently, the research results of Guan Hongzhi et al. also confirmed that the level of IgG in peripheral blood of patients with autoimmune encephalitis was high before immunotherapy, but gradually decreased during the treatment [34]. The above conclusions have also been reported in foreign studies [35]. These findings indicate that both humoral immunity and cellular immunity are involved in the pathogenesis of autoimmune encephalitis and are closely related to clinical efficacy.

The results showed that the level of IgG in cerebrospinal fluid of AE group was higher than that of VE group, and the level of IgM was lower than that of VE group. The difference was statistically significant, but there was no significant difference in the level of IgA between the two groups. It is suggested that there is intrathecal IgG synthesis and enhanced humoral immune response in AE group, which may be related to the abnormal increase of IgM caused by early virus infection in VE group. Plasma cells and lymphocytes in CSF can not only accelerate the increase of immunoglobulin synthesis in CSF, but also increase intrathecal immunoglobulin, especially IgG in CSF. Thus, when immunoglobulins are increased in the CSF, the degree of increase correlates with the degree of inflammation, suggesting that such patients are sensitive to immunosuppressive and first-line hormone therapy.

The MR images of AE and VE patients were further analyzed. The results showed that AE patients mostly involved the lateral ventricle, hippocampus, occipital lobe, and parietal lobe, VE patients mostly involved the frontal lobe and temporal lobe, and MS mostly involved the lateral ventricle and occipital lobe. The proportion of bilateral extensive lesions in both groups was significantly higher than 50%. The proportions of patients in the AE group involving the lateral ventricle, insula, and parietal lobes were significantly higher than those in the VE group, and the proportions involving the basal ganglia, temporal lobes, and frontal lobes were significantly lower than those in the VE group, and the differences were statistically significant. This has indicated that various lesions are involved in the MR images of both AE and VE patients, suggesting that clinical monitoring of such patients should be strengthened in order to promote the improvement of the patient’s condition. However, the involved parts of AE patients and VE patients are different, and the treatment should also vary from person to person and disease to disease.

The ROC curve was used to analyze the diagnostic value of these immune indexes in autoimmune encephalitis. The results showed that the AUC of IgG, IgA and IgM in the diagnosis of AE were 0.795, 0.602and 0.627respectively with the sensitivity values of 81.24%,65.63% and 53.15% and the specificity values of 65.08%, 57.54% and 75.01% respectively. The AUC of IgA + IgM in the diagnosis of AE was 0.733, and the sensitivity and specificity were 62.58% and 75.07% respectively. The AUC of IgA + IgG in the diagnosis of AE was 0.823, and the sensitivity and specificity were 81.24% and 67.54% respectively. The AUC of IgG + IgM in the diagnosis of AE was 0.886, and the sensitivity and specificity were 84.48% and 77.59% respectively. The AUC of IgA + IgM + IgG in the diagnosis of AE was 0.924, the sensitivity was 93.82%, and the specificity was 77.56%, which indicated that cerebrospinal fluid immunoglobulin had high application value in the diagnosis of autoimmune encephalitis in children, while the sensitivity of IgA + IgM + IgG combined diagnosis was the highest, which had more advantages than two-index combined diagnosis and single-index diagnosis. The application of IgA + IgM + IgG combined with diagnosis of AE can accurately determine the patient’s condition, and then provide guidance for follow-up treatment and contribute to the recovery of the patient’s condition.

When compared to demographic and clinical variables, age and BMI demonstrated no significant differences, exhibiting comparable mean values (AE Group: 5.27 ± 1.41 years, VE Group: 5.41 ± 1.37 years; AE Group: 23.10 ± 2.54 kg/m², VE Group: 23.06 ± 2.49 kg/m²). Based on the χ2 test (*P* > 0.05), gender distribution did not differ significantly between groups. The AE and VE groups had similar disease course durations (AE Group: 12.04 ± 3.87 days, VE Group: 11.87 ± 4.41 days, *P* > 0.05). Moreover, allergic illnesses and mR scores did not differ substantially between the two groups. The results showed that the AE and VE groups differed slightly regarding clinical symptoms. However, mental behaviour anomalies showed a little difference (AE Group: 70.83%, VE Group: 72.00%, χ2: 0.036, *P* > 0.05), but memory loss and the language barrier did not differ significantly. The differences between motor and sleep problems were not statistically significant. More preschool children were found in the AE Group compared to the VE Group (AE Group: 55.83%, VE Group: 55.00%, χ2: 0.015, *P* > 0.05). CSF immunoglobulin levels differed between AE and VE groups. IgG levels were substantially greater in the AE Group (35.73 ± 2.21 g/L) compared to the VE Group (18.26 ± 0.41 g/L) (t = 77.912, *P* < 0.05). However, IgA and IgM levels were similar between groups. Age and gender variations between cohorts with antibody-defined AIE subtypes were striking. While patients with AIE linked with GABABR, IgLON5, LGI1, CASPR2, and AMPAR antibodies had a median age of 60 years or older, those with GABAAR, DPPX, GAD, and GlyR antibodies were younger. The age distribution of GABAAR antibody patients was bimodal, with a very young group. Patients with NMDAR antibody-associated AIE were the youngest, averaging 27 years. Gender distributions varied among AIE subtypes, as did age. There was a moderate male predominance in AIE with DPPX, LGI1, and GABABR antibodies and a moderate female predominance in AMPAR, GABAAR, and NMDAR antibody-positive patients. Females were extremely rare in CASPR2 antibody-positive patients (14%), and males were extremely rare in GAD antibodies. In clinical neurology, AIE is considered in patients with new-onset epilepsy, mental illnesses, especially in younger individuals, and dementia or delirium in the elderly. Early detection of AIE may improve immunosuppressive therapy, but missed diagnosis may cause lifelong cognitive impairment. Thus, CSF data confirming the inflammatory origin of neurological sequelae play a part in the diagnostic criteria for AIE recently provided by numerous specialists. CSF in AIE sometimes lacks inflammatory alterations. It is possible that each antibody-defined AIE subtype has CSF results that reflect its immunological pathology. To corroborate this notion, we systematically examined CSF findings in published cases of 10 forms of AIE associated with well-defined antineuronal antibodies. Most individuals with individual results did not report all three CSF values. In addition, abnormal CSF cell numbers and levels of proteins were more likely to be reported, introducing significant bias, consequently, we only analysed pathological values to avoid bias from differential reporting of normal values among the 10 AIE subtypes. The normal cell count, CSF protein readings, and likely measurement methods differed slightly. There were no reports of CSF erythrocyte count, which may falsely raise CSF cell count. Most patients did not know the duration of the CSF analysis in relation to their diagnosis or immunosuppressive medication.

## Conclusion

To sum up, the level of immunoglobulin in spinal fluid is an effective reference index for clinical diagnosis of autoimmune encephalitis in children. When patients are admitted to hospital, MR examination and immunoglobulin level in cerebrospinal fluid should be perfected as soon as possible to diagnose the disease and be treated as soon as possible in order to improve their clinical prognosis. The deficiency of this study is that the sample size is relatively small, and large-sample clinical studies with scientific design, rigorous implementation and reliable results are still needed.

## Data Availability

The datasets used and/or analysed during the current study are available from the corresponding authors on reasonable request.
